# The Gut Microbiome and Metabolome of Domestic Cats Were Altered by the Oral Administration of Complex Probiotics

**DOI:** 10.3390/biology15080652

**Published:** 2026-04-20

**Authors:** Yanfeng Ma, Yuhua Hu, Junjie Zhang, Qing Sun, Hongyan Wang, Xinda Liu, Weipeng Tian, Wenhao Wang, Xuelian Ma, Donghua Shao, Ke Liu, Beibei Li, Yafeng Qiu, Zhiyong Ma, Zongjie Li, Jianchao Wei

**Affiliations:** 1 College of Agriculture, Jinhua University of Vocational Technology, Jinhua 321007, China; jhnxmyf@163.com (Y.M.); jhwanghy@163.com (H.W.); 2 Shanghai Veterinary Research Institute, Chinese Academy of Agricultural Science, Shanghai 200241, China; 13605398205@163.com (Y.H.); 17317271403@163.com (J.Z.); sun7355608@outlook.com (Q.S.); shaodonghua@shvri.ac.cn (D.S.); liuke@shvri.ac.cn (K.L.); lbb@shvri.ac.cn (B.L.); yafengq@shvri.ac.cn (Y.Q.); zhiyongma@shvri.ac.cn (Z.M.); 3 Pet Nutrition Research and Development Center, Gambol Pet Group Co., Ltd., Liaocheng 252000, China; liuxinda@gambolpet.com (X.L.); tianweipeng@gambolpet.com (W.T.); wangwenhao@gambolpet.com (W.W.); maxuelian@gambolpet.com (X.M.)

**Keywords:** domestic cat, complex probiotics, metagenomics, gut microbiome, metabolomics, intestinal metabolome

## Abstract

Probiotics are commonly applied to maintain the balance of gut microbiota and regulate the intestinal metabolic function of companion animals. In the present study, complex probiotics (*Bacillus coagulans* SNZ-1969, *Bacillus subtilis*, and *Bacillus licheniformis*) were added into the basal diet of domestic cats to investigate their influence on the intestinal microbiome and metabolic characteristics. Results revealed that the alpha diversity of the gut microbiota in the probiotic group was enhanced when compared to the control group. The beta diversity of the gut microbiota was also altered by the oral consumption of the complex probiotics. Compared to the control group, the relative abundance of beneficial microbes (such as *Clostridium*, *Bacteroides*, *Phocaeicola*, and *Ruminococcus*) in the probiotic group was enhanced, while the relative abundance of opportunistic pathogens (such as *Escherichia*, *Gallibacter*, *Corynebacterium*) was decreased. Additionally, the intestinal metabolic characteristics of domestic cats were also changed. The metabolomic analysis identified 408 differential metabolites between the two groups, and the KEGG function pathway analysis proved that the dominant pathway related to the differential metabolites were the amino acid metabolism, lipid metabolism, carbohydrate metabolism, energy metabolism, endocrine system, digestive system, immune system, and other metabolic pathways. Spearman’s correlation analysis revealed that the beneficial microbes had positive correlations with the differential metabolites. In conclusion, the current study demonstrated that oral administration of complex probiotics could regulate overall health and well-being in domestic cats through modulating the gut microbiome and metabolic characteristics.

## Simple Summary

The gut microbiota that harbor in the gastrointestinal tract of domestic cats play an important role in the occurrence and progression of systemic diseases. The current research revealed that consumption of complex probiotics could influence the alpha and beta diversity of the gut microbiota in domestic cats. By increasing the relative abundance of beneficial microbes and decreasing the relative abundance of opportunistic pathogens, the consumption of complex probiotics could regulate digestive function in domestic cats. A total of 408 differential metabolites were identified between the control and probiotic group through the metabolomic analysis, and there were 89 significantly up-regulated metabolites and 319 significantly down-regulated metabolites. The KEGG function pathway analysis revealed that the dominant pathway in the identified differential metabolites included the amino acid metabolism, lipid metabolism, carbohydrate metabolism, energy metabolism, endocrine system, digestive system, immune system, and other metabolic pathways. Therefore, oral administration of complex probiotics could regulate the gut microbial balance and protect the gastrointestinal function of domestic cats.

## 1. Introduction

Domestic cats have lived alongside humans for more than 10,000 years. Nowadays, domestic cats share the same living environment and a similar diet with their owners [1]. Pet cats are often regarded as family members and are closely integrated into their owner’s life, and intimate contact with pet cats can influence their owners’ physical and mental health [2,3]. Previous studies have shown that the furry pets played a critical role in transferring beneficial microbes, and the exchanged symbiotic microbiome may affect the pet owners’ physiology and decrease the risk of asthma, immune disorder, obesity, and other diseases [4,5]. Actually, frequent contact with companion animals can also help their owners to protect the gut barrier and improve immune function [6,7].

With the development of early agricultural activities, the modern domestic cats (*Felis silvestris catus*) were artificially domesticated from the Near Eastern wildcat (*Felis silvestris lybica*) [8,9]. In fact, the morphological, genetic, and behavioral characteristics of modern domestic cats are still quite similar to their wild ancestors. However, humans have markedly altered the food sources of domestic cats over the long process of artificial domestication. Although the wildcats are usually considered as obligate carnivores, the dietary structure of the pet cats has been gradually changed due to their owner’s choice [10,11]. The changed life-style of the domestic cats also influenced their intestinal metabolic function and gut microbiome composition.

Previous study has already proved that the core bacterial communities of the feline fecal microbiome were mainly composed of *Firmicutes*, *Proteobacteria*, *Actinobacteria*, *Bacteroidetes*, *Fusobacteria*, and others [12]. Compared to the traditional moderate protein diets, the high-protein/low-carbohydrate diets (HPLC) increase the species richness and microbial diversity of kittens, as well as enhancing the relative abundance of *Clostridium*, *Faecalibacterium*, *Ruminococcus*, *Blautia*, and *Eubacterium* [13,14]. Aging can also influence the gastrointestinal microbiota of domestic cats and increase the incidence of chronic diseases (such as Type 2 diabetes and enteropathies) [15,16]. The microbial communities of fecal microbiota in cats with chronic enteropathies were different from those in the healthy cats. When compared to the healthy cats, the relative abundance of *Bacteroides*, *Bifidobacterium*, *Chiranonis*, *Faecalibacterium* and *Turicibacter* were significantly decreased, while the relative abundance of *E. coli* and *Streptococcus* were significantly increased in cats with chronic enteropathies [17]. Moreover, the bacterial communities of gut microbiota in domestic cats also appear to differ among different breeds. Beneficial microbes (e.g., *Lactobacillus*, *Enterococcus*, *Streptococcus*, *Blautia*, *Roseburia*) are observed in the gut microbiota of healthy growing cats [18]. Therefore, the bacterial communities of the fecal microbiome are closely associated with the health of domestic cats.

Oral administration of probiotics can modulate the gut microbial balance and regulate the host’s nutritional absorption and metabolic functions [1,2]. Supplementations of single strain and/or the defined probiotic combinations can prevent and control the acute gastroenteritis, intestinal inflammation, respiratory disease, allergy, and other diseases of companion animals [19,20,21]. Recently, advances in high-throughput sequencing technology have already facilitated the research on the beneficial effects of pet probiotics, especially for their regulating effects on nutrient absorption and metabolism [22,23]. The beneficial effects of pet probiotics are mainly associated with the production of functional metabolites, such as short-chain fatty acids (SCFAs), serotonin, gamma-aminobutyric acid (GABA), indoles and other anti-inflammatory compounds [24,25]. Therefore, consumptions of probiotics can help modulate the immune system, reduce stress, protect against pathogenic bacteria, and promote the integral health of pet cats [26]. However, the specific action mechanism of the pet probiotic is still unclear. Perhaps the positive effects of complex probiotics are associated with gut microbiota composition and intestinal metabolites. In this study, the influence of complex probiotics on the gut microbiota and metabolic characteristics of domestic cats were analyzed.

## 2. Materials and Methods

### 2.1. Animals

A total of 16 healthy domestic cats (6 male and 10 female) ranging from 2 to 4 years of age were used in the current study. They were owned by Shanghai Veterinary Research Institute (SHVRI), Chinese Academy of Agricultural Sciences. All the cats were housed at the Centre for Feline Nutrition in a separate cabinet with elevated platforms for climbing and enrichment toys, and had ad libitum access to diet and clean water throughout the study. Normal physical examination was performed by a board-certified veterinary internal medicine specialist to confirm the absence of respiratory and gastrointestinal diseases and lack of infection or inflammation and the cats’ health and welfare were concerned throughout the whole study stage [15,18,21]. The animal experiment was approved by the experimental animal administration and ethics committee of SHVRI (number: SV-20241227-15).

### 2.2. Probiotics Administration

The domestic cats were randomly divided into two groups (the control group and the probiotic group), with each group containing 3 male and 5 female cats (*n* = 8). After a 2-week washout period, all the domestic cats were continued to be fed for a 4-week trial period. The control group received a basic diet with nutrient composition provided by the manufacturer (Gambol Pet Group Co., Ltd., Liaocheng, China) (Table 1). The probiotic group received complex probiotics (containing approximately 6 × 10^9^ CFU/kg of *Bacillus coagulans* SNZ-1969, 4 × 10^9^ CFU/kg of *Bacillus subtilis*, and 2 × 10^9^ CFU/kg of *Bacillus licheniformis*) according to the previous studies with proper modifications [20,21,27]. Both diets were formulated to meet the nutrient requirements for growth, gestation and lactation.

### 2.3. Sample Collection

According to the previously described methods, the fresh fecal specimens from domestic cats were collected after oral administration of complex probiotics for 4 weeks [21,27]. The collected fecal samples were added into 5 mL sterile tubes and were stored at −80 °C for further use.

### 2.4. Fecal Microbial Profiling

Metagenomics sequencing was conducted to analyze the diversity and structure of the gut microbiota. Briefly, total genomic DNA was extracted and the quality was verified by agarose gel electrophoresis, and then was determined with a NanoDrop2000 (Thermo Fisher Scientific, Waltham, MA, USA). A paired-end library was constructed using NEXTFLEX^®^ Rapid DNA-Seq (Bioo Scientific, Austin, TX, USA). Paired-end sequencing was performed on an Illumina NovaSeq (Illumina Inc., San Diego, CA, USA) at Majorbio Bio-Pharm Technology Co., Ltd. (Shanghai, China). The data were analyzed on the Majorbio Cloud Platform (www.majorbio.com). After quality control, high-quality paired-end reads were generated and assembled into nonredundant gut microbial genes. Metagenomics data were assembled using MEGAHIT (https://github.com/voutcn/megahit, version 1.1.2) (accessed on 18 March 2025). Representative sequences of nonredundant gene catalog were aligned to the NR database using Diamond (https://github.com/bbuchfink/diamond, version 0.8.35) (accessed on 18 March 2025) for taxonomic annotations [28,29,30,31].

### 2.5. Intestinal Metabolomics Analysis

The microbial metabolites in the cat fecal samples were extracted according to the previous study with proper modifications [32,33]. All the samples were ground using a Wonbio-96c frozen tissue grinder for 6 min (−10 °C, 50 Hz), and then they were extracted using low-temperature ultrasonic for 30 min (5 °C, 40 kHz). The samples were left at −20 °C for 30 min, and then the mixture was centrifuged for 15 min (4 °C, 13,000× *g*). Finally, the supernatant was collected, and the LC-MS/MS analysis was performed using a UHPLC-Q Exactive HF-X system equipped with an ACQUITY HSS T3 column at Majorbio Bio-Pharm Technology Co., Ltd. (Shanghai, China). The mass spectrometer scanned at an ion-spray voltage floating (ISVF) of −2800 V in negative mode and 3500 V in positive mode. The raw data of LC/MS was treated with a Progenesis QI software v2.3 (Waters Corporation, Milford, CT, USA). The peak extraction, peak alignment, deconvolution, and normalization of the downstream data were performed. The metabolites were identified by searching the HMDB (http://www.hmdb.ca/) and Metlin (https://metlin.scripps.edu/) databases. Spearman’s correlation heatmap was generated to analyze the specific correlations between the differential metabolites and the microbial communities [34,35,36].

### 2.6. Statistical Analysis

All the results are presented as the mean  ±  SD of at least triplicate measurements with *p*-values  <  0.05 considered statistically significant. Alpha diversity indices of microbial communities between the two groups were compared using a two-tailed Wilcoxon rank-sum test. The differences in beta diversity analysis between the two groups were compared by the permutational multivariate analysis of variance (PERMANOVA) with analysis of similarities (ANOSIM). The differential metabolites were determined using the variable importance in the projection (VIP) obtained by the orthogonal least partial squares discriminant analysis (OPLS-DA) and the *p*-value generated by Student’s *t* test. The screening criteria for differential metabolites was (VIP) > 1 and *p* < 0.05.

## 3. Results

### 3.1. The Basal Health State of the Enrolled Domestic Cats

All the domestic cats were weighed on days 0 and 28, and the consumed feed was measured and calculated. As shown in Table 2, there were no significant differences in the average daily feed intake and the body weight between the two groups. The current results indicated that the diet meets the nutrient requirements for the domestic cats.

### 3.2. Analyses of the Gut Microbial Diversities in the Domestic Cats

**Figure 1:**
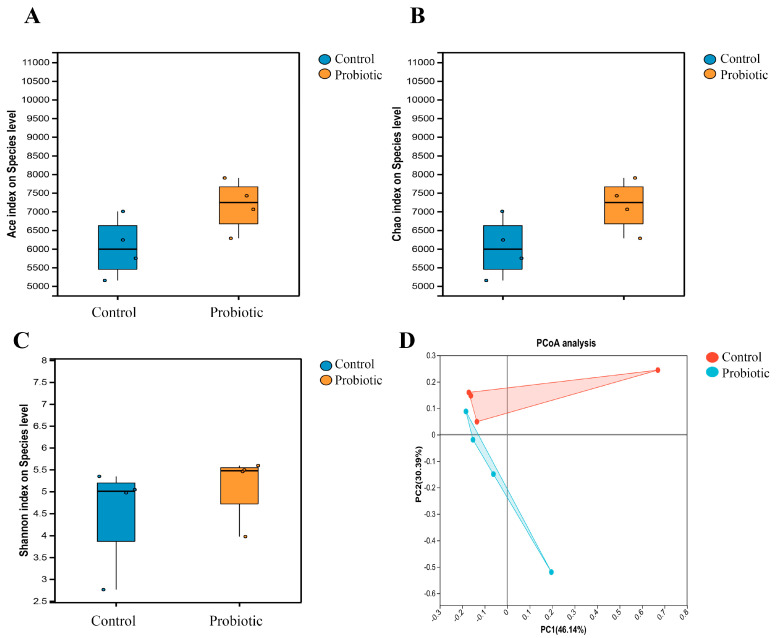
The alpha and beta diversities of the gut microbiota in the domestic cats. The Ace and Chao indices indicated that the bacterial richness of the probiotic group was much higher than the control group (**A**,**B**), and the Shannon index demonstrated that the bacterial diversity of the probiotic group was also much higher than the control group (**C**). PCoA analysis indicated that the microbial communities of the control and probiotic groups were segregated into different clusters (**D**).

In the current study, the alpha and beta diversity of the gut microbiota in the domestic cats which were affected by the oral administration of the complex probiotics were both analyzed. For the alpha diversity analysis, the Ace and Chao indices were used to indicate the bacterial richness, while the Shannon index was used to indicate the bacterial diversity. As shown in Figure 1A,B, the bacterial richness in the probiotic group was higher than that in the control group. The Shannon index demonstrated that the bacterial diversity in the probiotic group was also much higher than that in the control group (Figure 1). The current results indicated that the alpha diversity of gut microbiota in the domestic cats was increased by the oral administration of the complex probiotics. Moreover, the principal coordinate analysis (PCoA) was applied for the beta diversity. As shown in Figure 1D, the microbial communities of the control and probiotic groups were segregated into different clusters, indicating that the beta diversity of the gut microbiota in the domestic cats was changed by the oral administration of the complex probiotics.

### 3.3. Analyses of the Gut Microbial Compositions in the Domestic Cats

**Figure 2:**
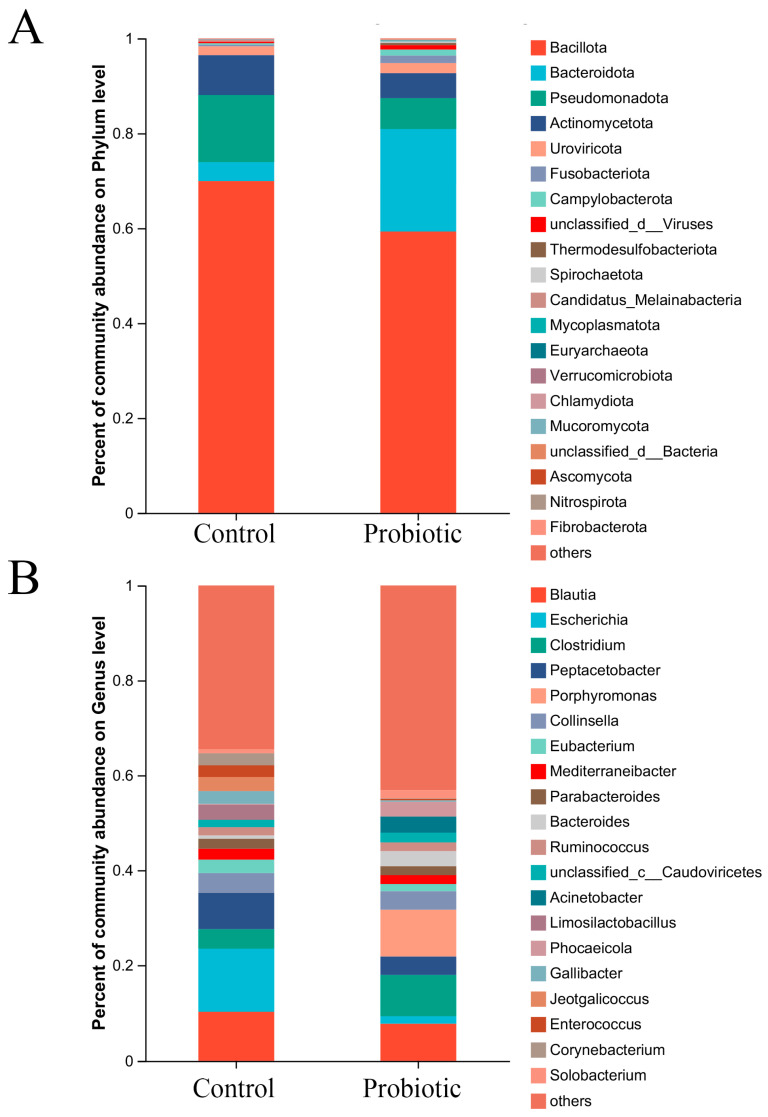
The microbial compositions at the phylum (**A**) and genus (**B**) levels. The microbial compositions of the control and probiotic groups were analyzed and compared, respectively.

The microbial compositions of the control and probiotic groups were analyzed and compared at the phylum and genus levels, respectively. The most dominant phyla were composed of Bacillota, Bacteroidota, Pseudomonadota, Actinomycetota, Uroviricota, and others (Figure 2A).

At the phylum level, the relative abundance of Bacteroidota in the probiotic group (21.62%) was higher than that in the control group (3.99%), however the relative abundance of Pseudomonadota in the probiotic group (6.49%) was lower than that in the control group (14.15%). At the genus level, the most dominant genera of the control and probiotic groups were composed of *Blautia* (10.26% vs. 7.76%), *Escherichia* (13.20% vs. 1.57%), *Clostridium* (4.11% vs. 8.62%), *Peptacetobacter* (7.68% vs. 3.85%), *Porphyromonas* (0.00% vs. 9.86%), *Collinsella* (4.13% vs. 3.91%), *Eubacterium* (2.84% vs. 1.50%), *Mediterraneibacter* (2.29% vs. 1.87%), *Parabacteroides* (2.11% vs. 1.89%), *Bacteroides* (0.71% vs. 3.81%), *Ruminococcus* (1.73% vs. 1.85%), *unclassified_c__Caudoviricetes* (1.53% vs. 2.03%), *Acinetobacter* (0.01% vs. 3.40%), *Limosilactobacillus* (3.20% vs. 0.01%), *Phocaeicola* (0.16% vs. 3.10%), *Gallibacter* (2.71%vs. 0.31%), *Jeotgalicoccus* (2.96% vs. 0.01%), *Enterococcus* (2.47%vs 0.33%), *Solobacterium* (0.85% vs. 1.71%), *Corynebacterium* (2.54% vs. 0.11%), and others (Figure 2B). As shown in Table 3, the relative abundance of beneficial microbes (e.g., *Clostridium*, *Bacteroides*, *Phocaeicola*, and *Ruminococcus*) in the probiotic group were higher than the control group. However, the relative abundance of opportunistic pathogens (e.g., *Escherichia*, *Gallibacter*, and *Corynebacterium*) in the probiotic group were lower than in the control group. Therefore, the current results revealed that oral administration of the complex probiotics could change the domestic cat’s microbial composition both at the phylum and genus levels.

### 3.4. Analyses of the Gut Microbial Metabolites in the Domestic Cats

**Figure 3:**
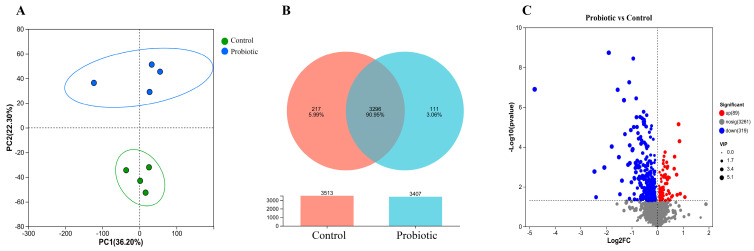
Untargeted metabolomics analysis of the differential metabolites in the two groups. Principal component analysis (PCA) revealed that the microbial metabolites of the two groups were classified into two different clusters (**A**). Venn diagram demonstrated the shared and unique microbial metabolites between the two groups (**B**). Volcano plot analysis of the differential metabolites. The red and blue dots represented up-and down-regulated metabolites, respectively (**C**).

With the aim to identify the differential metabolites between the control group and the probiotic group, principal component analysis (PCA) was performed. As shown in Figure 3A, the microbial metabolites of the two groups formed two distinct clusters. A Venn diagram was constructed to illustrate the shared and unique metabolites between the two groups. In total, there were 3296 shared microbial metabolites between the two groups. There were still 217 unique microbial metabolites in the control group and 111 unique microbial metabolites in the probiotic group (Figure 3B). The differential metabolites between the two groups were further analyzed using the volcano map. Compared to the control group, there were 89 significantly up-regulated metabolites and 319 significantly down-regulated metabolites in the probiotic group (Figure 3C). All the differential metabolites were mainly composed of amino acids, peptides, analogs, carbohydrates and carbohydrate conjugates, bile acids, alcohols and derivatives, fatty acids and conjugates, bilirubins, indoles, indolines, indolyl carboxylic acids and derivatives, quaternary ammonium salts, purine 2″-deoxyribonucleosides, purines and purine derivatives, alpha hydroxy acids and derivatives, gamma butyrolactones, carbonyl compounds, carboxylic acid derivatives, and others (Table S1). These results demonstrated that oral administration of the complex probiotics had changed the metabolic characteristics of the domestic cats.

**Figure 4:**
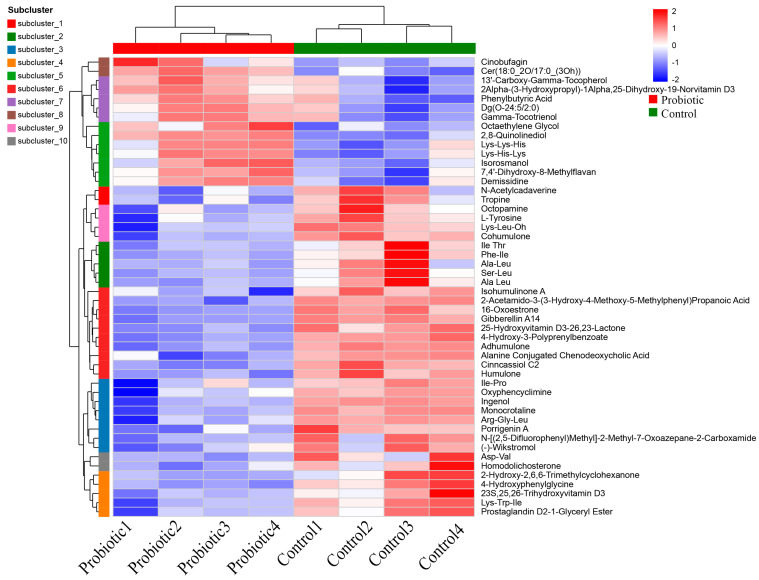
Metabolomics analysis for the hierarchical cluster diagram. The differential metabolites between the two groups were identified.

A total of 408 differential metabolites between the two groups were identified by the metabolomic analysis of the hierarchical clustering, and the top 50 of differential metabolites between the two groups were divided into ten subclusters (Figure 4).

### 3.5. KEGG Function Pathway and Enrichment Analyses

**Figure 5:**
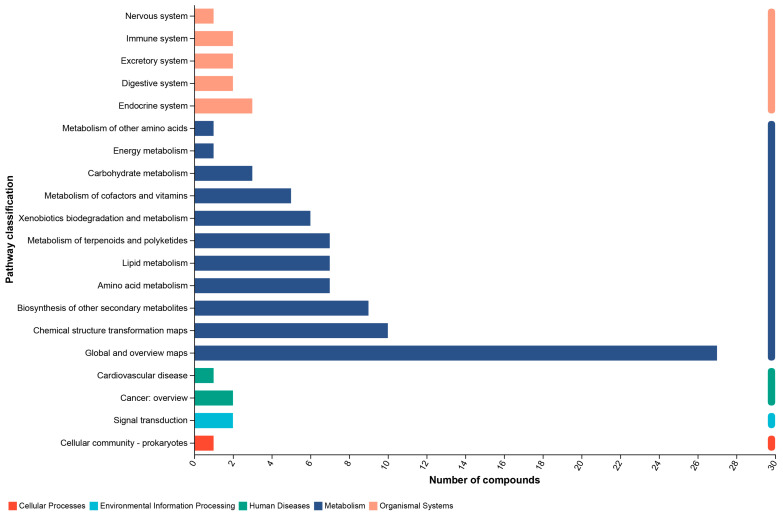
The KEGG function pathway analysis. The most dominant pathways were identified.

The Kyoto encyclopedia of genes and genomes (KEGG) function pathway analysis revealed that the dominant pathway included the amino acid metabolism, lipid metabolism, carbohydrate metabolism, energy metabolism, endocrine system, digestive system, immune system, and other metabolic pathways (Figure 5).

**Figure 6:**
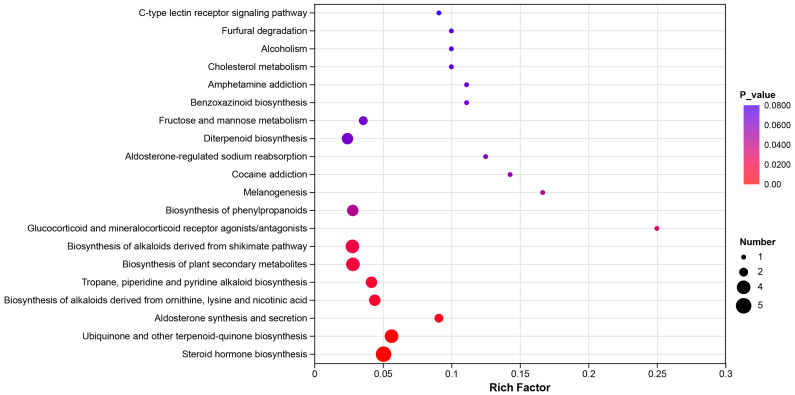
The KEGG enrichment analysis demonstrated the most influenced pathway between the two groups. The circle size revealed the impact value of metabolite enrichment in the pathway.

Moreover, the KEGG enrichment analysis indicted that the most influenced pathway included steroid hormone biosynthesis, aldosterone synthesis and secretion, tropane, piperidine and pyridine alkaloid biosynthesis, biosynthesis of alkaloids derived from ornithine, lysine and nicotinic acid, aldosterone-regulated sodium reabsorption, biosynthesis of phenylpropanoids, fructose and mannose metabolism, cholesterol metabolism, furfural degradation, C-type lectin receptor signaling pathway, and other metabolic pathways (Figure 6). In detail, the up-regulated pathway included the C-type lectin receptor signaling pathway, cholesterol metabolism, benzoxazinoid biosynthesis, and fructose and mannose metabolism. Conversely, the down-regulated pathway included furfural degradation, glucocorticoid and mineralocorticoid receptor agonists/antagonists, aldosterone-regulated sodium reabsorption, melanogenesis, and other pathways. The current results indicated that oral administration of the complex probiotics could alter the metabolic profiles of domestic cats.

### 3.6. Correlation Between Gut Microbiota and Metabolites

**Figure 7:**
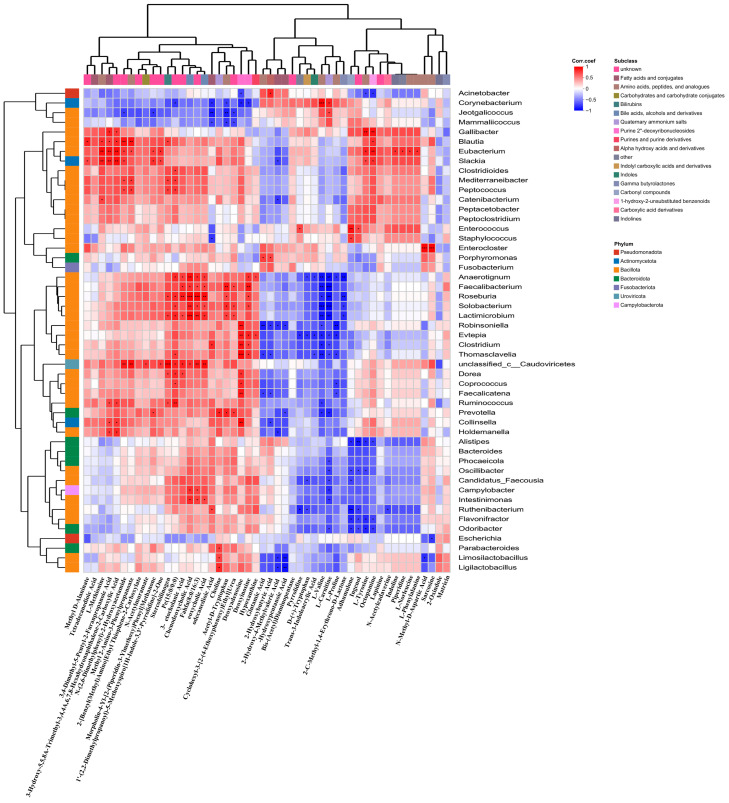
Spearman’s correlation heatmap between differential metabolites and microbial communities. Darker colors indicated stronger correlations. * *p* < 0.05, ** *p* < 0.01, *** *p* < 0.001.

To analyze the special correlations between the differential metabolites and the microbial communities, Spearman’s correlation heatmap was generated at the genus level (shown in Figure 7). Several genera of beneficial microbes (including *Clostridium*, *Ruminococcus*, *Faecalibacterium*, *Roseburia*, *Anaerotignum*, *Prevotella*, *Eubacterium*, *Slackia*, and *Blautia*) were found to have positive correlation with the differential metabolites. As shown in Figure 7, *Clostridium* was found to be positively correlated with Deoxyadenosine, Dodecanedioic Acid, and Deoxyinosine. *Faecalibacterium* was found to be positively correlated with Deoxyinosine, N-Acetyl-D-Tryptophan, Chenodeoxycholic Acid, 3-Ketocholanic Acid, and Deoxycholic Acid. *Roseburia* was found to be positively correlated with Chenodeoxycholic Acid, Deoxycholic Acid, 3-Ketocholanic Acid, Stercobilinogen, Deoxyinosine, and N-Acetyl-D-Tryptophan. *Eubacterium* was found to be positively correlated with L-Methionine, Carboxylic Acid, Octopamine, 3,4-Dimethyl-5-Pentyl-2-Furanpropanoic Acid, L-Tyrosine, L-Norleucine, L-Phenylalanine, Piperidine, Indoline, N-(2,6-Dimethylphenyl)-2-Hydroxyacetamide, Morpholin-4-Yl-[2-(Piperidin-3-Ylmethoxy) Phenyl] Methanone, and Methyl 2-Amino-3-Phenylpropanoate. *Slackia* was found to be positively correlated with 3-Hydroxy-5,5,8A-Trimethyl-3,4,4A,6,7,8-Hexahydronaphthalene-2-Carboxylic Acid, L-Methionine,3,4-Dimethyl-5-Pentyl-2-Furanpropanoic Acid, Morpholin-4-Yl-[2-(Piperidin-3-Ylmethoxy)Phenyl]Methanone, Octopamine, N-(2,6-Dimethylphenyl)-2-Hydroxyacetamide, 1′-(2,2-Dimethylpropanoyl)-5-Methoxyspiro [1H-Indole-3,3′-Pyrrolid-ine]-2-One, L-Tyrosine, and Methyl D-Alaninate. The differential metabolites which were found to be positively correlated with the microbial communities were associated with the amino acid metabolism, lipid metabolism, carbohydrate metabolism, energy metabolism, endocrine system, digestive system, and immune system.

## 4. Discussion

The gut microbes that were harbored in the mammalian gastrointestinal tract are intimately associated with the host’s health, even being taken as an invisible “organ”. The gut microbiota plays an important role in the onset and progression of systemic diseases, including inflammatory bowel disease, allergies, obesity, stress symptoms, and other disorders [1,37]. By producing several kinds of nutritional metabolites (such as amino acids, bile acids, and SCFAs), the gut microbiota can modulate the domestic cat’s immune system and provide protective benefits against pathogens [38,39]. Recent studies have shown that administration of complex probiotics helps the domestic cats establish a stable microbial ecosystem and maintain immune homeostasis [40,41,42].

In the present study, we investigated the influence of complex probiotics on the intestinal microbiome and metabolic characteristics of domestic cats. Consistent with previous research, the alpha diversity of the gut microbiota in the probiotic group was higher than those in the control group. As shown in Figure 1, the bacterial richness and diversity of the probiotic group was much higher than those of the control group. In fact, the numbers of bacteria in the stomach, small intestine, colon, and feces of domestic cats are quite different [26]. Supplementations of pet probiotics could enhance the richness and diversity of the intestinal microbiota through multiple kinds of action mechanisms [27]. For example, the applied pet probiotics could colonize in the gastrointestinal tracts and form colonization resistance to inhibit the growth of pathogens [43]. Moreover, probiotics could also alter the domestic cat’s gut microbiota by interacting with the host intestinal cells [2]. Simultaneously, PCoA results demonstrated that the microbial communities of the two groups were segregated into different clusters, indicating that the beta diversity of the gut microbiota in the domestic cats was also altered by the oral administration of the complex probiotics (Figure 1D). Therefore, application of probiotics could enhance the gut epithelial barrier and maintain the immune homeostasis through influencing the alpha and beta diversity of the intestinal microbiota.

Consumption of complex probiotics also altered the bacterial communities of the gut microbiota in the domestic cats. The *B. coagulans* SNZ-1969 strain is a rod-shaped and spore-forming Gram-positive bacteria with high safety and efficacy [44,45,46]. Moreover, *B. subtilis* and *B. licheniformis* are also broadly used to maintain the gut microbiota balance and protect the intestinal barrier function [43]. In fact, dietary supplementation of *B. licheniformis* and *B. subtilis* can also improve cat immunity and maintain intestinal health [47]. Multistrains of complex probiotics (*B. coagulans* SNZ 1969, *B. clausii* SNZ 1971, and *B. subtilis* SNZ 1972) were certified to be able to improve several symptoms of gastrointestinal discomfort [48]. In the current study, the relative abundance of Bacteroidota in the probiotic group was enhanced when compared to the control group (Figure 2A). Previous study had proved that *Firmicutes, Bacteroidetes, Fusobacteria, Proteobacteria and Actinobacteria* were the most predominant phyla in the fecal microbiota of domestic cats. The members of *Bacteroidetes* were certified to be intimately associated with nutrient digestibility and energy utilization [26]. However, the relative abundance of Pseudomonadota in the probiotic group was decreased when compared to the control group (Figure 2A). Certain members of Pseudomonadota might cause gut microbial dysbiosis and lead to intestinal diseases, such as diarrhea, inflammatory bowel disorders, and chronic enteropathies [26]. At the genus level, the relative abundance of beneficial microbes (such as *Clostridium*, *Bacteroides*, *Phocaeicola*, and *Ruminococcus*) in the probiotic group was higher than the control group (Figure 2B). Conversely, the relative abundance of opportunistic pathogens (such as *Escherichia*, *Gallibacter*, *Corynebacterium*) in the probiotic group was lower than the control group (Figure 2B). The current results revealed that oral administration of the complex probiotics could regulate microbial composition in the domestic cats. The enhanced percentage of beneficial microbes could promote the domestic cat’s health by producing lactic acid, SCFAs, bacteriocins, and other antimicrobial molecules [24,25]. Therefore, consumption of probiotics demonstrated positive effects on the cat’s immune system by increasing immunoglobulin levels and inhibiting the inflammatory factors expression [42].

The domestic cats are usually considered as strict carnivores that rely on high-protein diets to fulfill their nutritional requirements. However, the interactions of probiotics with fiber, starch, and protein content have strong effects on their intestinal metabolome [12]. Oral administration of complex probiotics also altered the metabolic characteristics of domestic cats. As shown in Figure 3A, PCA revealed that the microbial metabolites of the control group and the probiotic group formed distinct clusters. The Venn analysis demonstrated that a total of 3296 shared microbial metabolites between the two groups were identified, and there were still 217 unique microbial metabolites in the control group and 111 unique microbial metabolites in the probiotic group, respectively (Figure 3B). The volcano plot identified 89 significantly up-regulated metabolites and 319 significantly down-regulated metabolites (Figure 3C). The metabolomic analysis of the hierarchical clustering identified 408 differential metabolites between the two groups (Figure 4). Previous study demonstrated that dietary supplementation with *B. licheniformis* and *B. subtilis* could regulate the levels of L-Glycine and Sn-Glycero-3-phosphocholine in cat serum [47]. Oral administration of the probiotics could regulate cat intestinal metabolism by producing many useful metabolites, such as SCFAs, bacteriocins, indoles, secreted proteins, and extracellular vesicles. Moreover, consumption of probiotics could also maintain intestinal microecological balance and protect the intestinal epithelial barrier by increasing the secretion of antimicrobial peptides and enhancing the expression of tight junction proteins [2,42]. In the present study, the identified differential metabolites were mainly composed of 1-hydroxy-2-unsubstituted benzenoids, alcohols and polyols, amino acids, peptides, and analogs, benzoic acids and derivatives, carbohydrates and carbohydrate conjugates, ergostane steroids, fatty acyl glycosides, halobenzenes, hydroxysteroids, pregnane steroids, purines and purine derivatives, sesquiterpenoids, terpene glycosides, and others. Therefore, the supplementation of complex probiotics could alter intestinal metabolic profiles of the domestic cats.

Supplementations of complex probiotics could improve the energy metabolism and regulate the intestinal immune system through modulating the gut microbiota composition. Moreover, optimization of the gut microbiota could also help the domestic cats to enhance nutrient digestion and absorption [49]. KEGG function pathway analysis revealed that the dominant pathways in the identified differential metabolites were associated with the amino acid metabolism, lipid metabolism, carbohydrate metabolism, energy metabolism, endocrine system, digestive system, immune system, and other metabolic pathways (Figure 5). KEGG enrichment analysis indicated that the most influenced pathways between the two groups were steroid hormone biosynthesis, aldosterone synthesis and secretion, tropane, piperidine and pyridine alkaloid biosynthesis, biosynthesis of alkaloids derived from ornithine, lysine and nicotinic acid, aldosterone-regulated sodium reabsorption, biosynthesis of phenylpropanoids, fructose and mannose metabolism, cholesterol metabolism, furfural degradation, C-type lectin receptor signaling pathway, and other metabolic pathways (Figure 6). In fact, supplementations of the complex probiotics could promote the domestic cat’s intestinal health by producing various kinds of bacterial metabolites, such as SCFAs, bile acids, and amino acids [50]. Consumption of complex probiotics could ferment the dietary fibers and produce acetate, propionate, and butyrate, and these bacterial metabolites could protect the epithelial barrier and suppress the gut inflammation [51]. Because the synthesis of arginine and taurine is very limited, the gut microbiota could help the domestic cats to maintain the amino acid balance through the digestion of high-quality dietary protein [52]. Moreover, the gut microbiota could help the domestic cats to transfer the primary bile acids into secondary bile acids through the deconjugation and dihydroxylation [53]. Therefore, the positive effects of complex probiotics could help to maintain the intestinal homeostasis, improve the gut barrier function, and modulate the immune system.

Spearman correlation analysis was performed to examine the associations between the differential metabolites and the microbial communities (Figure 7). Beneficial microbes (including *Clostridium*, *Ruminococcus*, *Faecalibacterium*, *Roseburia*, *Anaerotignum*, *Prevotella*, *Eubacterium*, *Slackia*, and *Blautia*) were proved to have positive correlations with the differential metabolites. These differential metabolites could influence the amino acid metabolism, lipid metabolism, carbohydrate metabolism, energy metabolism, endocrine system, digestive system, and immune system.

## 5. Conclusions

In all, supplementation of complex probiotics demonstrated obvious regulating effects on the health and well-being of domestic cats through the modulation of the gut microbiome and metabolic characteristics. To date, the research on the fecal microbiome and metabolome of domestic cats is lacking. Therefore, a large amount of multi-omics research on the domestic cats’ intestinal characteristics are still needed to be performed to further explain the specific action mode and underlying mechanism of complex probiotics.

## Figures and Tables

**Table 1:** Primary ingredients of the utilized diets.

Ingredients	Nutrient Composition
Crude protein	46.0%
Crude fat	18.0%
Crude fiber	5.0%
Crude ash	10.0%
Calcium	1.0%
Phosphorus	0.8%
Taurine	0.3%
Chloride	0.3%
Moisture	10.0%
Energy	15,787 kJ/kg

**Table 2:** Measurements of the average daily feed intake and body weight.

Item	Average Daily Feed Intake (g)		Average Body Weight (kg)	
	Start	End	Start	End
Control	85.16 ± 5.61	85.12 ± 6.57	2.71 ± 0.23	2.78 ± 0.38
Probiotics	89.48 ± 4.21	87.40 ± 3.18	3.15 ± 0.55	3.08 ± 0.46

The data are shown as the mean ± S.E.M.

**Table 3:** The predominant taxonomic profiles of feline gut microbiota.

Taxonomic Level	Taxa	Average	Control	Probiotic
Phylum	Bacillota	64.61%	69.92%	59.29%
	Bacteroidota	12.81%	3.99%	21.62%
	Pseudomonadota	10.32%	14.15%	6.49%
	Actinomycetota	6.85%	8.41%	5.29%
	Uroviricota	1.99%	1.86%	2.12%
Genus	*Blautia*	9.01%	10.26%	7.76%
	*Escherichia*	7.38%	13.20%	1.57%
	*Clostridium*	6.37%	4.11%	8.62%
	*Peptacetobacter*	5.76%	7.68%	3.85%
	*Porphyromonas*	4.93%	0.00%	9.86%
	*Collinsella*	4.02%	4.13%	3.91%
	*Eubacterium*	2.17%	2.84%	1.50%
	*Mediterraneibacter*	2.08%	2.29%	1.87%
	*Parabacteroides*	2.00%	2.11%	1.89%
	*Bacteroides*	1.95%	0.71%	3.18%
	*Ruminococcus*	1.79%	1.73%	1.85%
	*Caudoviricetes*	1.78%	1.53%	2.03%
	*Acinetobacter*	1.71%	0.01%	3.40%
	*Limosilactobacillus*	1.61%	3.20%	0.01%
	*Phocaeicola*	1.63%	0.16%	3.10%
	*Gallibacter*	1.50%	2.70%	0.31%
	*Jeotgalicoccus*	1.49%	2.96%	0.01%
	*Enterococcus*	1.40%	2.47%	0.33%
	*Corynebacterium*	1.33%	2.54%	0.11%
	*Solobacterium*	1.28%	0.85%	1.71%
	Others	38.81%	34.51%	43.12%

## Data Availability

The raw reads were deposited into the NCBI Sequence Read Archive (SRA) database (Accession Number: PRJNA1376267).

## Supplementary Materials

The following supporting information can be downloaded at: https://www.mdpi.com/article/10.3390/biology15080652/s1, Table S1: Differential metabolites.

## References

[1] Grześkowiak Ł., Endo A., Beasley S., Salminen S. (2015). Microbiota and Probiotics in Canine and Feline Welfare. Anaerobe.

[2] Yang Q., Wu Z. (2023). Gut Probiotics and Health of Dogs and Cats: Benefits, Applications, and Underlying Mechanisms. Microorganisms.

[3] Gupta S. (2017). Microbiome: Puppy Power. Nature.

[4] Deng P., Swanson K.S. (2015). Gut Microbiota of Humans, Dogs and Cats: Current Knowledge and Future Opportunities and Challenges. Br. J. Nutr..

[5] Nermes M., Endo A., Aarnio J., Salminen S., Isolauri E. (2015). Furry Pets Modulate Gut Microbiota Composition in Infants at Risk for Allergic Disease. J. Allergy Clin. Immunol..

[6] Johnson C.C., Ownby D.R. (2017). The Infant Gut Bacterial Microbiota and Risk of Pediatric Asthma and Allergic Diseases. Transl. Res..

[7] Kil D.Y., Swanson K.S. (2011). Companion Animals Symposium: Role of Microbes in Canine and Feline Health. J. Anim. Sci..

[8] Driscoll C.A., Macdonald D.W., O’Brien S.J. (2009). From Wild Animals to Domestic Pets, an Evolutionary View of Domestication. Proc. Natl. Acad. Sci. USA.

[9] Krajcarz M., Krajcarz M.T., Baca M., Baumann C., Van Neer W., Popović D., Sudoł-Procyk M., Wach B., Wilczyński J., Wojenka M. (2020). Ancestors of Domestic Cats in Neolithic Central Europe: Isotopic Evidence of a Synanthropic Diet. Proc. Natl. Acad. Sci. USA.

[10] Haruda A.F., Ventresca Miller A.R., Paijmans J.L.A., Barlow A., Tazhekeyev A., Bilalov S., Hesse Y., Preick M., King T., Thomas R. (2020). The Earliest Domestic Cat on the Silk Road. Sci. Rep..

[11] Hu Y., Hu S., Wang W., Wu X., Marshall F.B., Chen X., Hou L., Wang C. (2014). Earliest Evidence for Commensal Processes of Cat Domestication. Proc. Natl. Acad. Sci. USA.

[12] Pilla R., Suchodolski J.S. (2021). The Gut Microbiome of Dogs and Cats, and the Influence of Diet. Vet. Clin. N. Am. Small Anim. Pract..

[13] Hooda S., Vester Boler B.M., Kerr K.R., Dowd S.E., Swanson K.S. (2013). The Gut Microbiome of Kittens Is Affected by Dietary Protein:Carbohydrate Ratio and Associated with Blood Metabolite and Hormone Concentrations. Br. J. Nutr..

[14] Deusch O., O’Flynn C., Colyer A., Morris P., Allaway D., Jones P.G., Swanson K.S. (2014). Deep Illumina-Based Shotgun Sequencing Reveals Dietary Effects on the Structure and Function of the Fecal Microbiome of Growing Kittens. PLoS ONE.

[15] Bermingham E.N., Young W., Butowski C.F., Moon C.D., Maclean P.H., Rosendale D., Cave N.J., Thomas D.G. (2018). The Fecal Microbiota in the Domestic Cat (*Felis catus*) Is Influenced by Interactions Between Age and Diet; A Five Year Longitudinal Study. Front. Microbiol..

[16] Sung C.H., Marsilio S., Pilla R., Wu Y.A., Cavasin J.P., Hong M.P., Suchodolski J.S. (2024). Temporal Variability of the Dominant Fecal Microbiota in Healthy Adult Cats. Vet. Sci..

[17] Sung C.H., Marsilio S., Chow B., Zornow K.A., Slovak J.E., Pilla R., Lidbury J.A., Steiner J.M., Park S.Y., Hong M.P. (2022). Dysbiosis Index to Evaluate the Fecal Microbiota in Healthy Cats and Cats with Chronic Enteropathies. J. Feline Med. Surg..

[18] Li Z., Di D., Sun Q., Yao X., Wei J., Li B., Liu K., Shao D., Qiu Y., Liu H. (2022). Comparative Analyses of the Gut Microbiota in Growing Ragdoll Cats and Felinae Cats. Animals.

[19] Belà B., Di Simone D., Pignataro G., Fusaro I., Gramenzi A. (2024). Effects of *L. reuteri* NBF 2 DSM 32264 Consumption on the Body Weight, Body Condition Score, Fecal Parameters, and Intestinal Microbiota of Healthy Persian Cats. Vet. Sci..

[20] Zhu S., Zha M., Xia Y. (2025). Complex Probiotics Suppress Inflammation by Regulating Intestinal Metabolites in Kittens. Animals.

[21] Vientós-Plotts A.I., Ericsson A.C., Rindt H., Reinero C.R. (2017). Oral Probiotics Alter Healthy Feline Respiratory Microbiota. Front. Microbiol..

[22] Alessandri G., Argentini C., Milani C., Turroni F., Cristina Ossiprandi M., van Sinderen D., Ventura M. (2020). Catching a Glimpse of the Bacterial Gut Community of Companion Animals: A Canine and Feline Perspective. Microb. Biotechnol..

[23] Suchodolski J.S. (2022). Analysis of the Gut Microbiome in Dogs and Cats. Vet. Clin. Pathol..

[24] Liu Y., Wang J., Zheng H., Xin J., Zhong Z., Liu H., Fu H., Zhou Z., Qiu X., Peng G. (2024). Multi-Functional Properties of Lactic Acid Bacteria Strains Derived from Canine Feces. Front. Vet. Sci..

[25] Wang J., Yang X., Peng Y., Zhang J., Huang Y., Zhong Z., Liu H., Fu H., Zhou Z., Peng G. (2024). Isolation and in vitro Investigation on Lactic Acid Bacteria for Potential Probiotic Properties from Cat Feces. Front. Vet. Sci..

[26] Lee D., Goh T.W., Kang M.G., Choi H.J., Yeo S.Y., Yang J., Huh C.S., Kim Y.Y., Kim Y. (2022). Perspectives and Advances in Probiotics and the Gut Microbiome in Companion Animals. J. Anim. Sci. Technol..

[27] Li Y., Ali I., Lei Z., Li Y., Yang M., Yang C., Li L. (2023). Effect of a Multistrain Probiotic on Feline Gut Health through the Fecal Microbiota and Its Metabolite SCFAs. Metabolites.

[28] Wu H., Lv B., Zhi L., Shao Y., Liu X., Mitteregger M., Chakaroun R., Tremaroli V., Hazen S.L., Wang R. (2025). Microbiome-Metabolome Dynamics Associated with Impaired Glucose Control and Responses to Lifestyle Changes. Nat. Med..

[29] Lin D., Chen X., Lin X., Zhang C., Liang T., Zheng L., Xu Y., Huang L., Qiao Q., Xiong K. (2025). New Insight into Intestinal Toxicity Accelerated by Aged Microplastics with Triclosan: Inflammation Regulation by Gut Microbiota-Bile Acid Axis. J. Hazard. Mater..

[30] Feng Y., Li L., Ma Q., Liu S., Wang P., Li X., Ma J. (2025). Effect of Microcystin-LR on Intestinal Microbiota, Metabolism, and Health of Zebrafish (*Danio rerio*). Sci. Total Environ..

[31] Han Y., Liu X., Jia Q., Xu J., Shi J., Li X., Xie G., Zhao X., He K. (2024). Longitudinal Multi-Omics Analysis Uncovers the Altered Landscape of Gut Microbiota and Plasma Metabolome in Response to High Altitude. Microbiome.

[32] Yang Y., Hao C., Jiao T., Yang Z., Li H., Zhang Y., Zhang W., Doherty M., Sun C., Yang T. (2025). Osteoarthritis Treatment via the GLP-1-Mediated Gut-Joint Axis Targets Intestinal FXR Signaling. Science.

[33] Zhao Y., Wang P., Wang D., Zhao W., Wang J., Ge Z., Liu Y., Zhao X. (2025). Gut Microbiota and Metabolic Profile Affected by Pectic Domains during in vitro Rat Fecal Fermentation: A Comparative Study between Different Glycans Rich in Pectic Monosaccharides. Carbohydr. Polym..

[34] Liu J., Liu Y., Huang C., He C., Yang T., Ren R., Xin Z., Wang X. (2025). Quercetin-Driven *Akkermansia Muciniphila* Alleviates Obesity by Modulating Bile Acid Metabolism via an ILA/m6A/CYP8B1 Signaling. Adv. Sci..

[35] Gao H., Sun M., Li A., Gu Q., Kang D., Feng Z., Li X., Wang X., Chen L., Yang H. (2025). Microbiota-Derived IPA Alleviates Intestinal Mucosal Inflammation through Upregulating Th1/Th17 Cell Apoptosis in Inflammatory Bowel Disease. Gut Microbes.

[36] Jin T., Li S.Y., Zheng H.L., Liu X.D., Huang Y., Ma G., Zhao Y.X., Zhao X.T., Yang L., Wang Q.H. (2025). Gut Microbes-Spinal Connection Is Required for Itch Sensation. Gut Microbes.

[37] Mondo E., Marliani G., Accorsi P.A., Cocchi M., Di Leone A. (2019). Role of Gut Microbiota in Dog and Cat’s Health and Diseases. Open Vet. J..

[38] Minamoto Y., Hooda S., Swanson K.S., Suchodolski J.S. (2012). Feline Gastrointestinal Microbiota. Anim. Health Res. Rev..

[39] Meazzi S., Stranieri A., Lauzi S., Bonsembiante F., Ferro S., Paltrinieri S., Giordano A. (2019). Feline Gut Microbiota Composition in Association with Feline Coronavirus Infection: A Pilot Study. Res. Vet. Sci..

[40] Sivamaruthi B.S., Kesika P., Chaiyasut C., Fukngoen P., Sisubalan N. (2025). A Review of Probiotic Supplementation and Its Impact on the Health and Well-Being of Domestic Cats. Vet. Sci..

[41] Xia J., Cui Y., Guo Y., Liu Y., Deng B., Han S. (2024). The Function of Probiotics and Prebiotics on Canine Intestinal Health and Their Evaluation Criteria. Microorganisms.

[42] Sun J., Gu X., Zhang H., Zhao L., Wang J., Wang X., Tao H., Wang Z., Han B. (2025). Application of Probiotics in Cats and Dogs: Benefits and Mechanisms. Vet. Sci..

[43] Sanders M.E., Merenstein D.J., Reid G., Gibson G.R., Rastall R.A. (2019). Probiotics and prebiotics in intestinal health and disease: From biology to the clinic. Nat. Rev. Gastroenterol. Hepatol..

[44] Metlakunta A.S., Soman R.J. (2020). Safety evaluation of *Bacillus coagulans* SNZ 1969 in Wistar rats. Regul. Toxicol. Pharmacol..

[45] Jung S.M., Ha A.W., Choi S.J., Kim S.Y., Kim W.K. (2022). Effect of *Bacillus coagulans* SNZ 1969 on the Improvement of Bowel Movement in Loperamide-Treated SD Rats. Nutrients.

[46] Murthy D.K., Soman R.J., Soman D., Pv K. (2025). Testing the Immunomodulatory Effects of Probiotic *Bacillus coagulans* SNZ 1969® in Healthy Adults: A Randomized, Double-Blind, Placebo-Controlled Trial. Cureus.

[47] Zhang M., Xu H., Zhang T., Kang J., Xu Z., Wu P., Niu Y., Shi Y., Zhong Y., Yang C. (2025). Dietary Supplementation with *Bacillus licheniformis* and *Bacillus subtilis* Modulates Immunity, Serum Metabolome, and Intestinal Homeostasis in Cats. Animals.

[48] Soman R.J., Swamy M.V. (2019). A prospective, randomized, double-blind, place-bo-controlled, parallel-group study to evaluate the efficacy and safety of SNZ TriBac, a three-strain Bacillus probiotic blend for undiagnosed gastrointestinal discomfort. Int. J. Colorectal Dis..

[49] de Vos W.M., Tilg H., Van Hul M., Cani P.D. (2022). Gut microbiome and health: Mechanistic insights. Gut.

[50] Gasaly N., de Vos P., Hermoso M.A. (2021). Impact of Bacterial Metabolites on Gut Barrier Function and Host Immunity: A Focus on Bacterial Metabolism and Its Relevance for Intestinal Inflammation. Front. Immunol..

[51] Lavelle A., Sokol H. (2020). Gut microbiota-derived metabolites as key actors in inflammatory bowel disease. Nat. Rev. Gastroenterol. Hepatol..

[52] Che D., Nyingwa P.S., Ralinala K.M., Maswanganye G.M.T., Wu G. (2021). Amino Acids in the Nutrition, Metabolism, and Health of Domestic Cats. Adv. Exp. Med. Biol..

[53] Collins S.L., Stine J.G., Bisanz J.E., Okafor C.D., Patterson A.D. (2023). Bile acids and the gut microbiota: Metabolic interactions and impacts on disease. Nat. Rev. Microbiol..

